# Supplementary material for: Extracellular Matrix Deposition and Remodeling after Corneal Alkali Burn in Mice
Source: Int J Mol Sci. 2021 May 27;22(11):5708. doi: 10.3390/ijms22115708 (PMC8199272; doi:10.3390/ijms22115708)
Supplement: Supplementary file 1 [file ijms-22-05708-s001.zip › Mutoji et al Supplementary data.pdf]

International Journal of *Molecular Sciences*

MDPI

Supplementary data

Extracellular matrix deposition and remodeling after corneal alkali burn in mice

Kazadi N. Mutoji[1], Mingxia Sun[1], Garrett Elliott[1], Isabel Y. Moreno[1], Clare Hughes[2], Tarsis F. Gesteira[1,3] and Vivien J. Coulson- Thomas[1,*]

1 College of Optometry, University of Houston, Houston, Texas, United States
2 School of Biosciences, Cardiff University
3 Optimvia
* Correspondence: Vivien J. Coulson-Thomas, University of Houston, College of Optometry, 4901 Calhoun Road, Houston, TX 77204-2020, USA; vcoulsonthomas@gmail.com or vjcoulso@central.uh.edu.

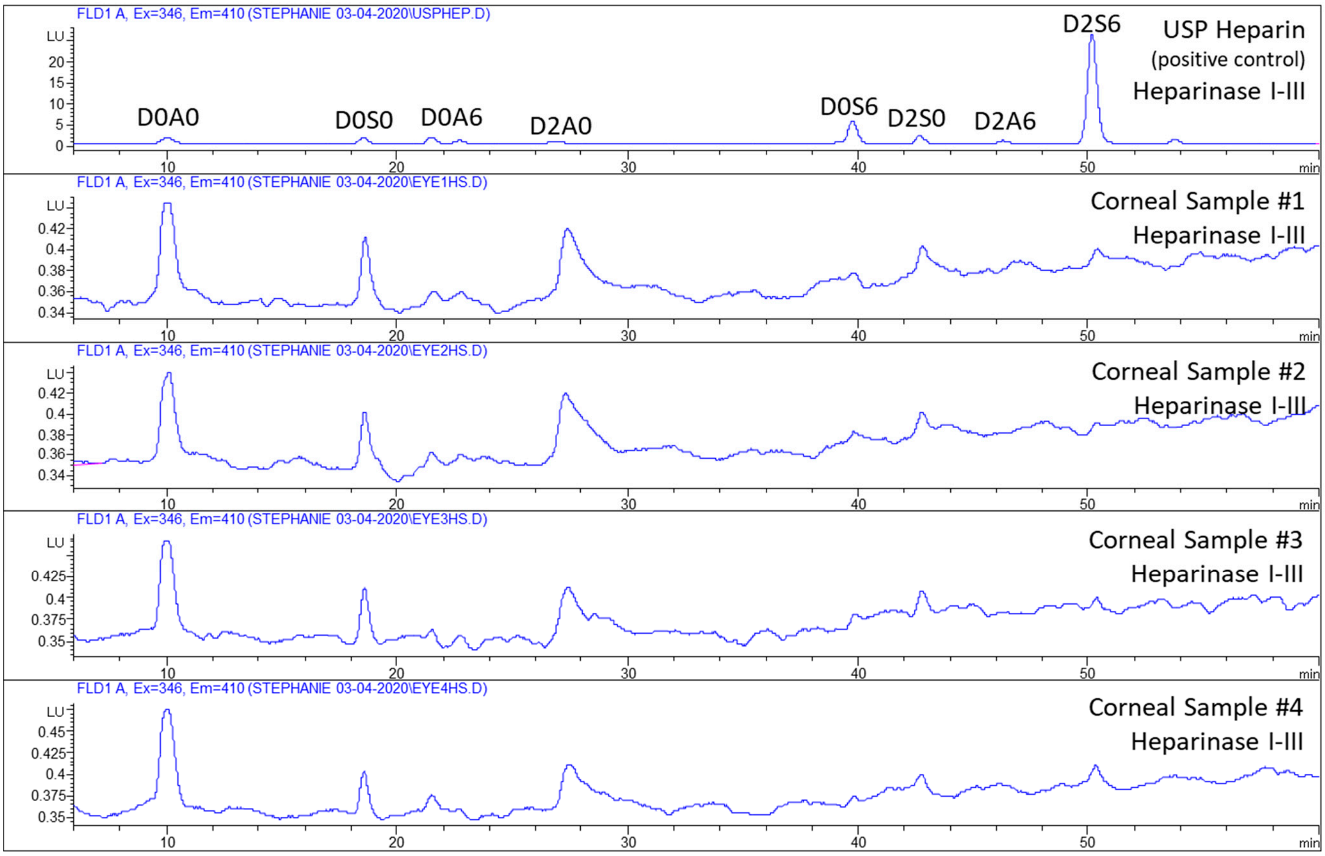

Supplemental Figure 1. Chromatogram of HS Standards and Corneal GAG Disaccharides Produced with Heparinase I-III. A representative chromatogram is evidenced for the HS analysis of corneas 5 and 14 days after AB compared to the contralateral control eye. Total GAGs were extracted from corneal tissues and digested with Heparinase I and III in order to generate HS disaccharides. These were then separated by strong anion exchange chromatography using a high pressure liquid chromatography system (SAX-HPLC). The eluent was subjected to fluorescent post-column derivatization. The disaccharide separation profiles were compared to the separation of standard disaccharides for identification and quantification of the different disaccharides in corneal HS (top chromatogram).

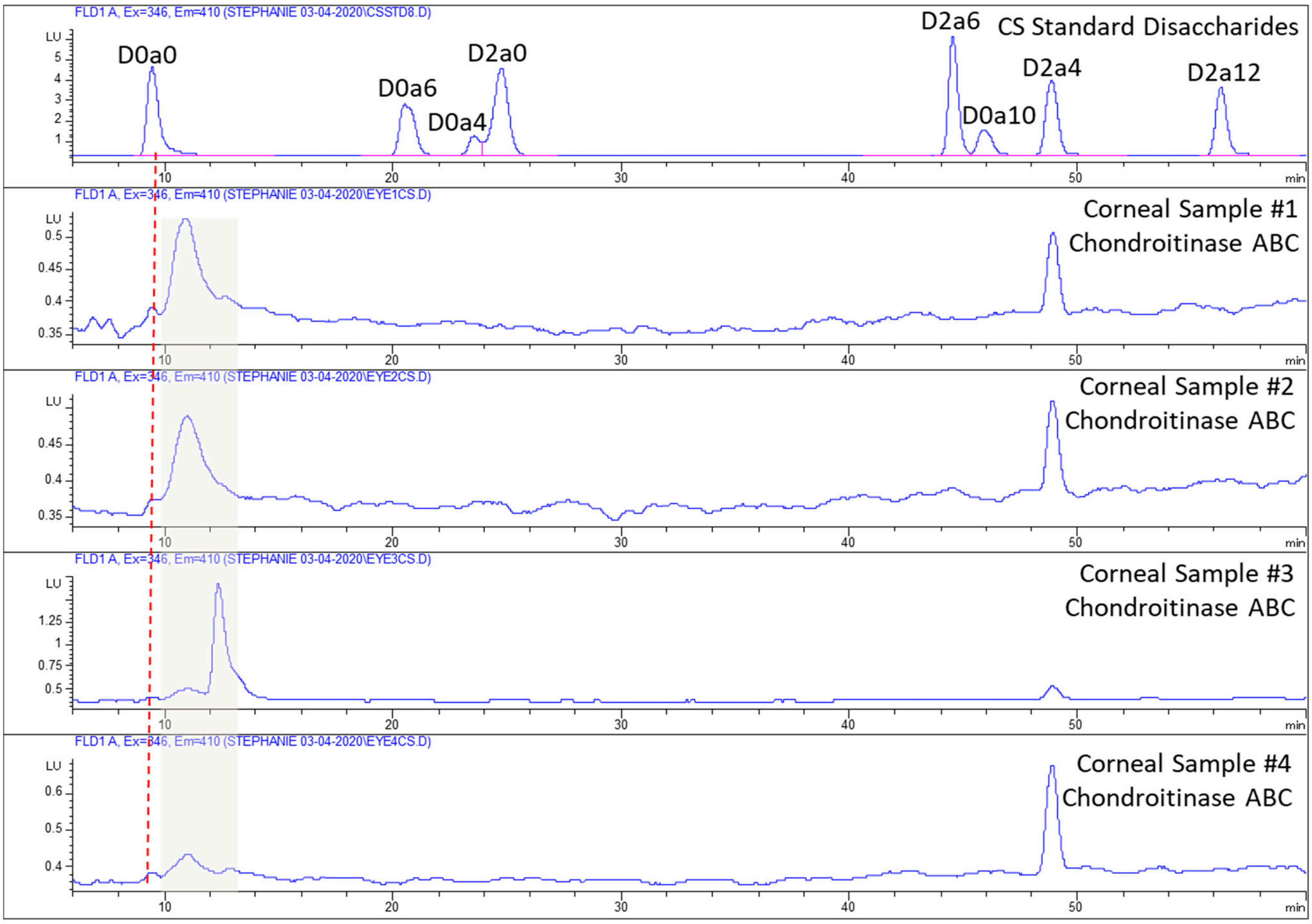

Supplemental Figure 2. Chromatogram of CS Standards and Corneal GAG Disaccharides Produced with Chondroitinase ABC. A representative chromatogram is evidenced for CS/DS analysis of corneas 5 and 14 days after AB compared to the contralateral control eye. Total GAGs were extracted from corneal tissues and digested with Chondroitinase ABC in order to generate CS/DS disaccharides. These were then separated by strong anion exchange chromatography using a high pressure liquid chromatography system (SAX-HPLC). The eluent was subjected to fluorescent post-column derivatization. The disaccharide separation profiles were compared to the separation of standard CS disaccharides for identification and quantification of the different disaccharides in corneal CS/DS (top chromatogram).
