# Supplementary figures and images for: Extracellular Matrix Deposition and Remodeling after Corneal Alkali Burn in Mice
Source: Int J Mol Sci. 2021 May 27;22(11):5708. doi: 10.3390/ijms22115708 (PMC8199272; doi:10.3390/ijms22115708)

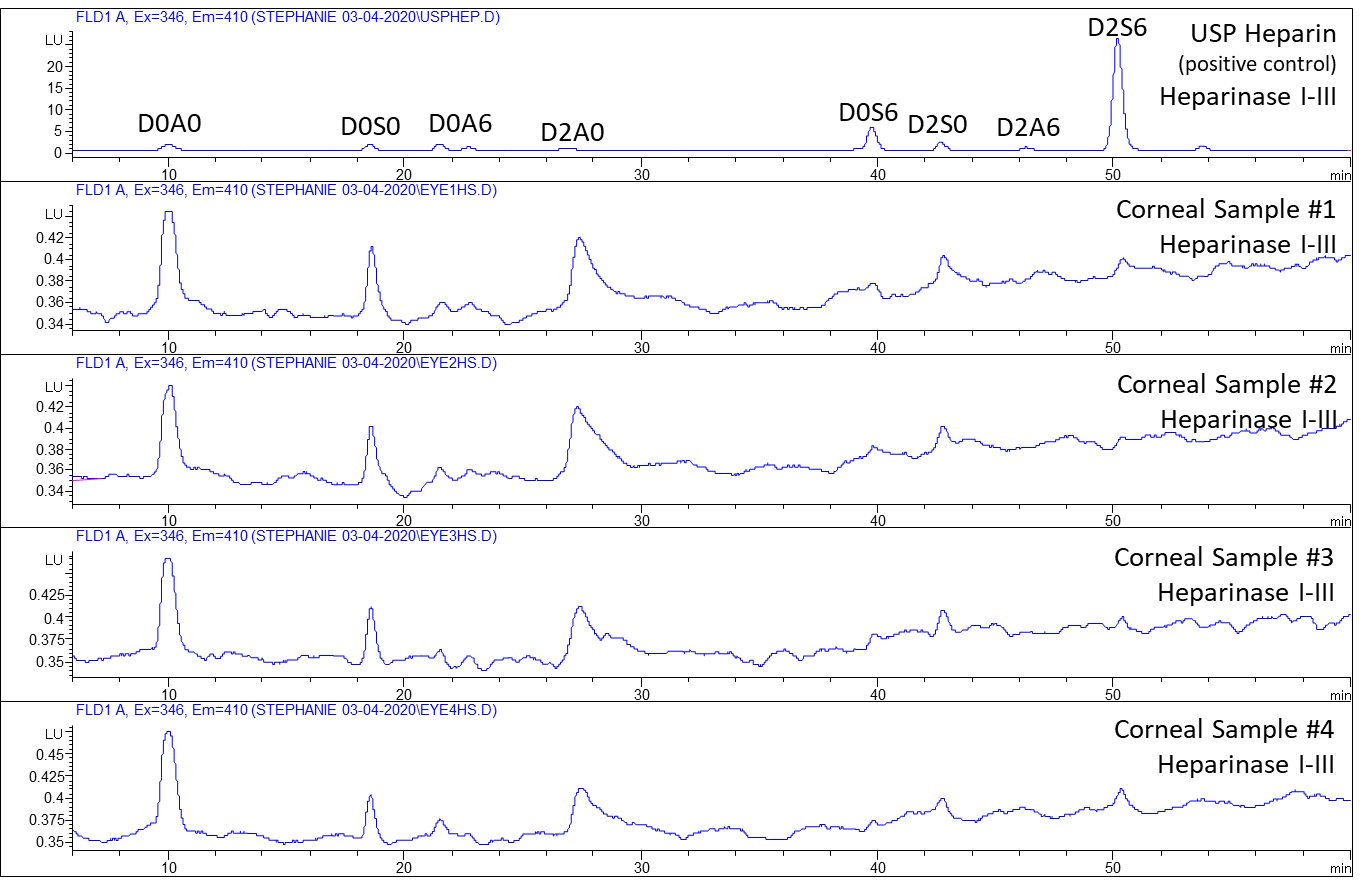

Supplement: Supplementary file 1 [file ijms-22-05708-s001.zip › Supplemental Figure 1.png]

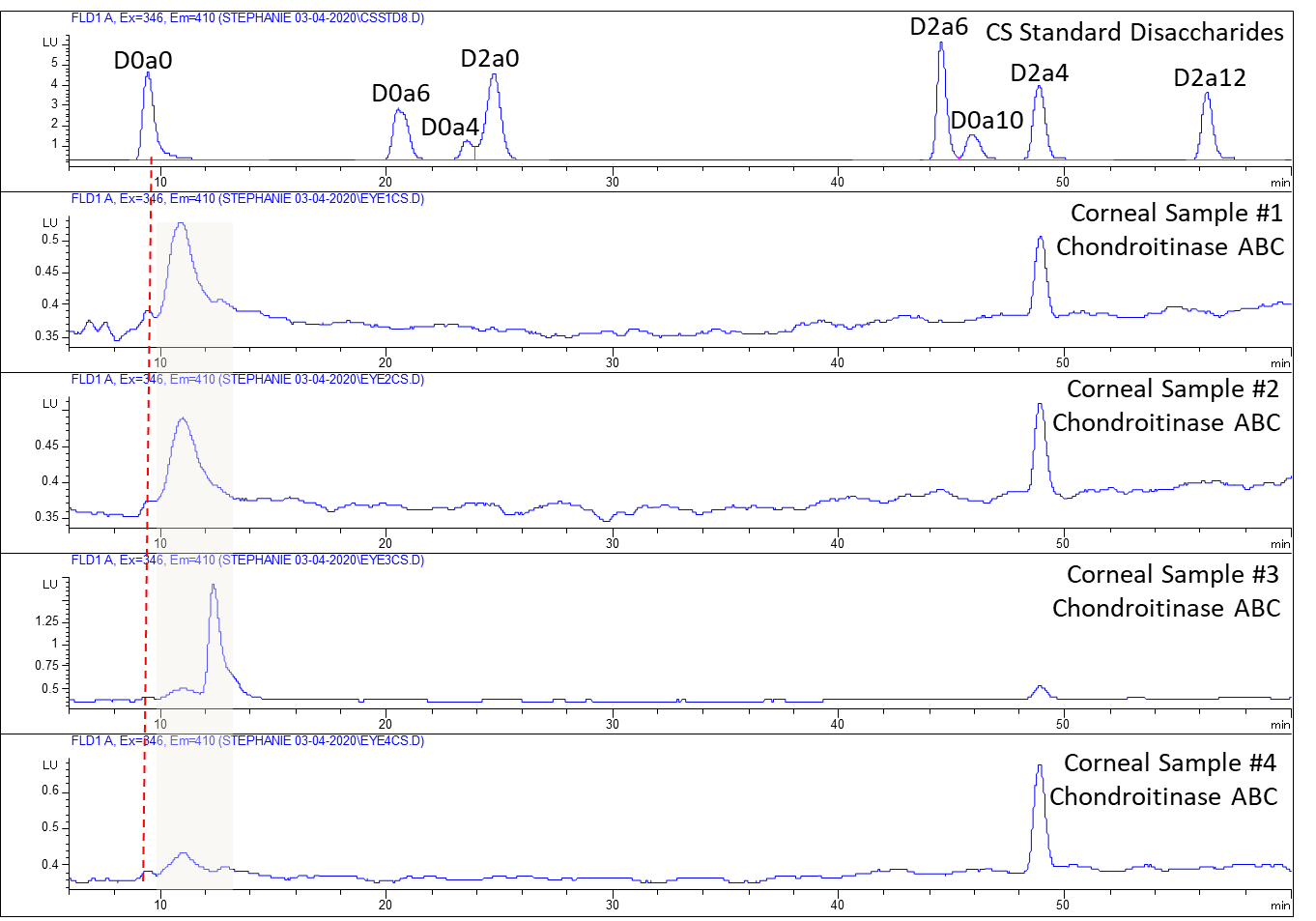

Supplement: Supplementary file 1 [file ijms-22-05708-s001.zip › Supplemental Figure 2.png]
